# PLC-gamma-1 phosphorylation status is prognostic of metastatic risk in patients with early-stage Luminal-A and -B breast cancer subtypes

**DOI:** 10.1186/s12885-019-5949-x

**Published:** 2019-07-30

**Authors:** Rossano Lattanzio, Manuela Iezzi, Gianluca Sala, Nicola Tinari, Marco Falasca, Saverio Alberti, Simonetta Buglioni, Marcella Mottolese, Letizia Perracchio, Pier Giorgio Natali, Mauro Piantelli

**Affiliations:** 1 Department of Medical, Oral and Biotechnological Sciences, ‘G. d’Annunzio’ University of Chieti–Pescara, Chieti, Italy; 2 Center for Advanced Studies and Technology (CAST), ‘G. d’Annunzio’ University of Chieti–Pescara, Via Luigi Polacchi 11, 66100 Chieti, Italy; 3 Department of Medicine and Aging Sciences, ‘G. d’Annunzio’ University of Chieti–Pescara, Chieti, Italy; 4 Metabolic Signalling Group, School of Pharmacy and Biomedical Sciences, Curtin Health Innovation Research Institute, Curtin University, Perth, Australia; 5 Department of Biotechnology BIOMORF, University of Messina, Via Consolare Valeria 1, 98125 Messina, Italy; 6 Department of Pathology, ‘Regina Elena’ National Cancer Institute, Via E. Chianesi, 53, 00144 Rome, Italy

**Keywords:** Breast cancer, Phospholipase Cγ1, Prognosis, Luminal subtypes, Menopausal status

## Abstract

**Background:**

Phospholipase Cγ1 (PLCγ1) is highly expressed in human tumours. Our previous studies reported that both stable and inducible PLCγ1 down-regulation can inhibit formation of breast-cancer-derived experimental lung metastasis. Further, high expression of PLCγ1 and its constitutively activated forms (i.e., PLCγ1-pY1253, PLCγ1-pY783) is associated with worse clinical outcome in terms of incidence of distant metastases, but not of local relapse in T1-T2, N0 breast cancer patients.

**Methods:**

In the present retrospective study, we analysed the prognostic role of PLCγ1 in early breast cancer patients stratified according to the St. Gallen criteria and to their menopausal status. PLCγ1-pY1253 and PLCγ1-pY783 protein expression levels were determined by immunohistochemistry on tissue microarrays, and were correlated with patients’ clinical data, using univariate and multivariate statistical analyses.

**Results:**

In our series, the prognostic value of PLCγ1 overexpression was restricted to Luminal type tumours. From multivariate analyses, pY1253-PLCγ1^High^ was an independent prognostic factor only in postmenopausal patients with Luminal-B tumours (hazard ratio [HR], 2.4; 95% confidence interval [CI], 1.1–5.3; *P* = 0.034). Conversely, PLCγ1-pY783^High^ was a remarkably strong risk factor (HR, 20.1; 95% CI, 2.2–178.4; *P* = 0.003) for pre/perimenopausal patients with Luminal-A tumours.

**Conclusions:**

PLCγ1 overexpression is a strong predictive surrogate marker of development of metastases in early Luminal-A and -B breast cancer patients, being able to discriminate patients with high and low risk of metastases. Therefore, targeting the PLCγ1 pathway can be considered of potential benefit for prevention of metastatic disease.

**Electronic supplementary material:**

The online version of this article (10.1186/s12885-019-5949-x) contains supplementary material, which is available to authorized users.

## Background

Breast cancer incidence accounts for approximately 30% of all new cancer cases and 14% of all cancer-related deaths among women worldwide [1]. Implementation of screening programmes can detect early stage, node-negative (N0) tumours at low-risk of relapse, while advances in adjuvant treatments can promote decreased mortality from breast cancer [2, 3]. Nevertheless, almost 80% of women diagnosed with early stage disease are currently treated by breast-conserving surgery alone or combined with adjuvant therapy [4]. In these patients without nodal metastasis (N0), identification of high-risk patients for breast cancer relapse through use of established prognostic factors (i.e., age, tumour size and grade, hormone receptor status) cannot predict prognosis accurately. Moreover, nearly 90% of patients with cancer limited to the breast receive adjuvant treatments, although only 30% of these patients will ultimately relapse [5–7]. Currently, adjuvant chemotherapy regimens are standard of care for treatment of early-stage disease that is oestrogen receptor (ER)-negative or human epidermal growth factor receptor (HER)2-positive. However, selecting chemotherapy for patients with ER-positive, HER2-negative disease is a more challenging task, due to the different risk profiles for disease relapse associated with this tumour subtype. Indeed, among patients with luminal tumours, there will be women at low risk of recurrence who will derive little benefit from chemotherapy combined with hormone therapy, and women at high-risk of recurrence where chemotherapy would be helpful. As far as the economic impacts on health care systems, is should be noted that adjuvant treatments are not devoid of toxicity. On this basis, above the search for new discriminatory biomarkers capable of selecting patients at risk of early relapse remains mandatory.

Among the lipid signalling metabolites in cells, the phosphoinositides are the most widely studied lipids due to their involvement in several cell signalling pathways. In particular, phosphoinositide-specific phospholipase C gamma 1 (PLCγ1) signalling is necessary for many physiological cellular processes (e.g., cell proliferation and differentiation) [8]. PLCγ1 is highly expressed in various tumours, including breast cancers [9–11]. We previously observed that PLCγ1 down-regulation strongly reduced formation of MDA-MB-231-derived lung metastases in nude mice [12]. Furthemore, lung metastasis formation from prostate cancer cells was significantly reduced by a dominant-negative fragment of PLCγ1 [13].

In human, using tumour cases as training and validation sets, we have shown that overexpression of activated PLCγ1 is a risk factor for distant relapse in T1-T2, N0 breast cancer patients undergoing adjuvant chemotherapy [14]. Therefore, in the present study, we investigated the prognostic role of PLCγ1 in these early breast cancer patients stratified according to the St. Gallen criteria and to their menopausal status.

## Methods

### Patients

We retrospectively reviewed the medical records of 979 consecutive patients (year range, 1995–2003) diagnosed with primary unilateral breast carcinoma at the “Regina Elena” National Cancer Institute, Rome, Italy. From the original series, only N0 patients with T1/T2 tumours were included in the present study (*n* = 414). The patients’ and tumour characteristics are given in Table 1.

**Table 1:** Patients and tumor characteristics (*n* = 414)

Variable	Value (%)
Age at diagnosis (yr)	
Median	59.7
< 50	103 (24.9)
50–65	173 (41.8)
> 65	138 (33.3)
Menopausal status	
Pre/perimenopausal	109 (26.3)
Postmenopausal	305 (73.7)
Molecular subtypes	
Luminal A	156 (37.7)
Luminal B	176 (42.5)
HER2	27 (6.5)
Triple negative	55 (13.3)
Tumour size	
≤ 2 cm	272 (65.7)
> 2 cm	142 (34.3)
Histotypes	
Ductal carcinoma	330 (79.7)
Lobular carcinoma	54 (13.0)
Other	30 (7.3)
Tumour grade	
1	64 (15.5)
2–3	350 (84.5)
ER	
Negative	89 (21.5)
Positive	325 (78.5)
PgR	
Negative	188 (45.4)
Positive	226 (54.6)
Ki-67	
Low	287 (69.3)
High	127 (30.7)
HER2	
Negative	348 (84.1)
Positive	66 (15.9)
PLCγ1	
Low	225 (54.3)
High	189 (45.7)
PLCγ1-pY1253	
Low	241 (58.2)
High	173 (41.8)
PLCγ1-pY783	
Low	327 (79.0)
High	87 (21.0)
Patient outcome	
Without recurrence	299 (72.2)
Local recurrence	50 (12.1)
Distant recurrence	65 (15.7)

This study was reviewed and approved by the Ethics Committee of the Regina Elena National Cancer Institute. All of the patients were treated with quadrantectomy and received radiation therapy (*n* = 414), while 172 received chemotherapy without or with hormonal therapy, and 160 underwent only hormonal therapy. Patients with HER2-positive tumours did not receive trastuzumab, because this immune treatment was not available during the study period. The median follow-up was 79 months (range, 2–298 months). Follow-up data were collected from institutional records or from the referring physicians. During follow-up, 50 patients (12.1%) experienced local relapse. Distant relapse was seen in 65 (15.7%) of the patients.

### Immunohistochemistry

The 414 breast cancer cases were distributed in 21 tissue microarrays (TMA) blocks assembled in duplicate. Briefly, TMAs were constructed by punching 2-mm-diameter cores of histologically proven invasive breast carcinoma areas, as previously described [14]. The tissue microarray sections were incubated with the mouse anti-PLCγ1 monoclonal antibody (sc-7290), and with the rabbit anti-PLCγ1-pY1253 (sc-22141-R) and anti-PLCγ1-pY783 (sc-12943-R) polyclonal antibodies, with all from Santa Cruz Biotechnology (Santa Cruz, CA, USA). Although these antibodies were validated by Santa Cruz Biotechnology, their specificities were further validated using appropriate silenced breast cancer cell lines (Additional file 2: Figure S1). The anti-mouse and anti-rabbit EnVision kits (Agilent, Santa Clara, CA, USA) were used for signal amplification, as appropriate. For the control tissues, the primary antibody was excluded or substituted with non-immune serum or isotype-matched immunoglobulins. The immunohistochemical analysis was carried out by two pathologists (R.L., M.P.) by agreement, with both blinded to the clinicopathological information. The immunohistochemical results for the ER, progesterone receptor (PgR), Ki67 and HER2 status were obtained from the patients’ hospital records.

### Statistical methods

The St. Gallen criteria [15] were used to dichotomise the tumour size and tumour grade, as well as the ER, PgR and Ki-67 protein expression. We also examined the distribution of the expression of PLCγ1 and its phosphorylated forms in four breast cancer molecular subtypes: Luminal-A (*n* = 156), Luminal-B (*n* = 176), HER2 (*n* = 27) and Triple Negative (*n* = 55). The expression of the PLCγ1, PLCγ1-pY1253 and PLCγ1-pY783 proteins were reported as percent of positive cells, and dichotomised (high vs. low) according to the cut-off values corresponding to the 50th (i.e., 75% of positive cells for PLCγ1 and 61% of positive cells for PLCγ1-pY1253) and 75th (i.e., 59% of positive cells for PLCγ1-pY783) percentiles, as previously defined [14]. In all immunohistochemical evaluations, interobserver agreement was scored as near-perfect (i.e., PLCγ1: kappa = 0.854; PLCγ1-pY1253: kappa = 0.861; PLCγ1-pY783: kappa = 0.889).

Pearson’s χ2 or Fisher’s exact tests were used to asssess the relations between the tumour PLCγ1, PLCγ1-pY1253 and PLCγ1-pY783 protein expression and the patient clinicopathological parameters. Disease-free survival (DFS) was defined as the interval from surgery to the first of the following events: tumour relapse at local or distant sites. Local relapse-free survival (LRFS) and distant relapse-free survival (DRFS) were defined as the time from surgery to the occurrence of local and distant relapses, respectively. Kaplan-Meier plots were used for the survival analyses, and log-rank tests were applied to compare the survival curves between the patient groups.

Cox’s proportional hazards models were used to evaluate the association of PLCγ1, PLCγ1-pY1253 and PLCγ1-pY783 expression with survival time, using covariates. The following covariates were computed in the multivariate models: tumour size, tumour grade, and ER, PgR, Ki-67, HER2, PLCγ1, PLCγ1-pY1253 and PLCγ1-pY783 status. The statistical software SPSS version 15.0 (SPSS, Chicago, IL, USA) was used throughout, and *P* < 0.05 was considered statistically significant.

## Results

### PLCγ1, PLCγ1-pY1253 and PLCγ1-pY783 immunostaining

**Fig. 1:**
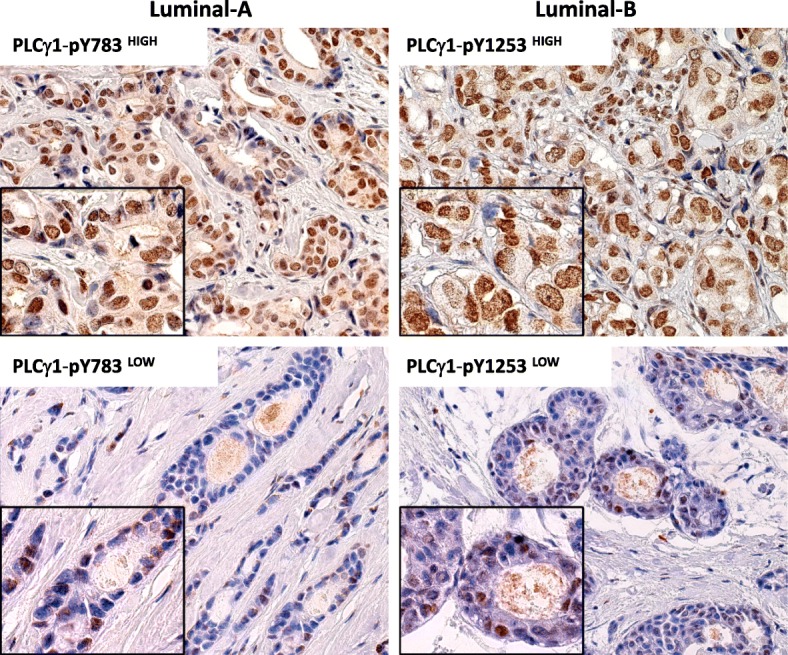
PLCγ1-pY1253 and PLCγ1-pY783 immunostaining: examples of high (upper panel) and low (lower panel) expression in Luminal-A and Luminal-B breast cancer subtypes. Expression of PLCγ1 phosphorylated forms is confined to tumor cell nuclei

As described previously [14], neoplastic cells presented cytoplasmic immunoreactivity for PLCγ1, whereas positivity for PLCγ1-pY1253 and PLCγ1-pY783 was almost exclusively nuclear (Fig. 1). In all, 189 of 414 (45.7%) patients showed high tumour PLCγ1 expression levels (PLCγ1^High^). Similarly for tumour expression of PLCγ1-pY1253^High^ and PLCγ1-pY783^High^, as 173/414 (41.8%) and 87/414 (21.0%), respectively (Table 1).

### Survival analysis in early breast cancer patients

#### All cases

Kaplan-Meier plots showed significant association of high tumour expression of PLCγ1, pY1253-PLCγ1 and pY783-PLCγ1 with low DFS rates (*P* = 0.005, *P* = 0.019, *P* = 0.006, respectively) (Additional file 2: Figure S2). In particular, tumours that overexpressed PLCγ1 or its activated forms were associated with significantly higher frequency of distant relapse (*P* = 0.001, *P* = 0.001, *P* = 0.005, respectively), while no significant correlations with local relapse were observed.

Multivariate analyses of DFS revealed prognostic significance of tumour expression of PLCγ1 (HR, 1.5; 95% CI, 1.0–2.3; *P* = 0.029), pY1253-PLCγ1 (HR, 1.6; 95% CI, 1.1–2.3; *P* = 0.024) and tumour grade (Additional file 1: Table S1). In addition, higher risk of distant but not of local relapse was seen for PLCγ1^High^ (HR, 2.1; 95% CI, 1.3–3.6; *P* = 0.005), pY1253-PLCγ1^High^ (HR, 2.3; 95% CI, 1.4–3.7; *P* = 0.001) and pY783-PLCγ1^High^ (HR, 1.7; 95% CI, 1.0–2.7; *P* = 0.049).

#### Tumour subtypes

**Fig. 2:**
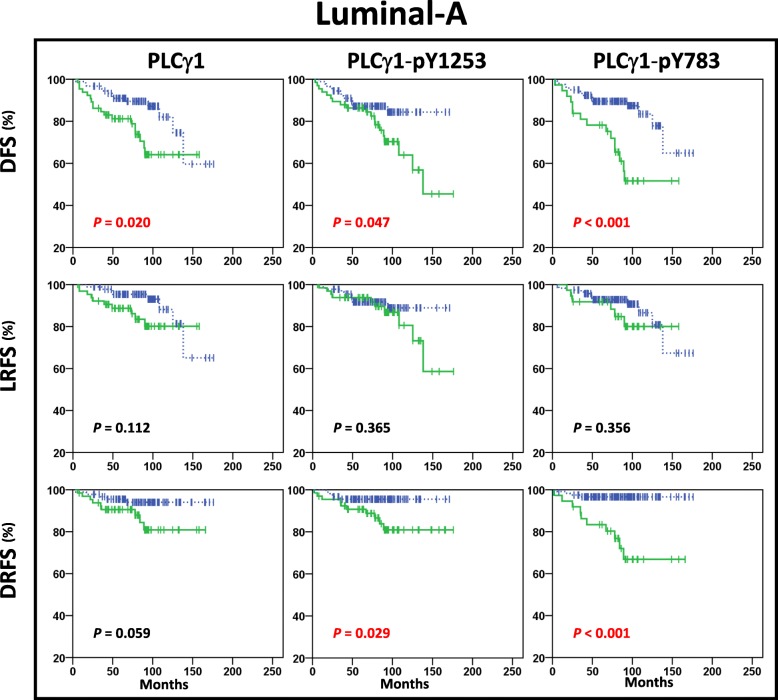
Kaplan-Meier plots in the Luminal-A subtype. Kaplan-Meier estimates of DFS, LRFS and DRFS in patients with Luminal-A tumours (*n* = 156), according to high (solid green lines) and low (dashed blue lines) expression of PLCγ1, PLCγ1-pY1253 and PLCγ1-pY783. In this cohort, the patients showed distant relapse in 6% (5/91) PLCγ1^Low^ and 14% (9/65) PLCγ1^High^, in 4% (4/90) PLCγ1-pY1253^Low^ and 15% (10/66) PLCγ1-pY1253^High^, and in 3% (4/119) PLCγ1-pY783^Low^ and 27% (10/37) PLCγ1-pY783^High^

**Fig. 3:**
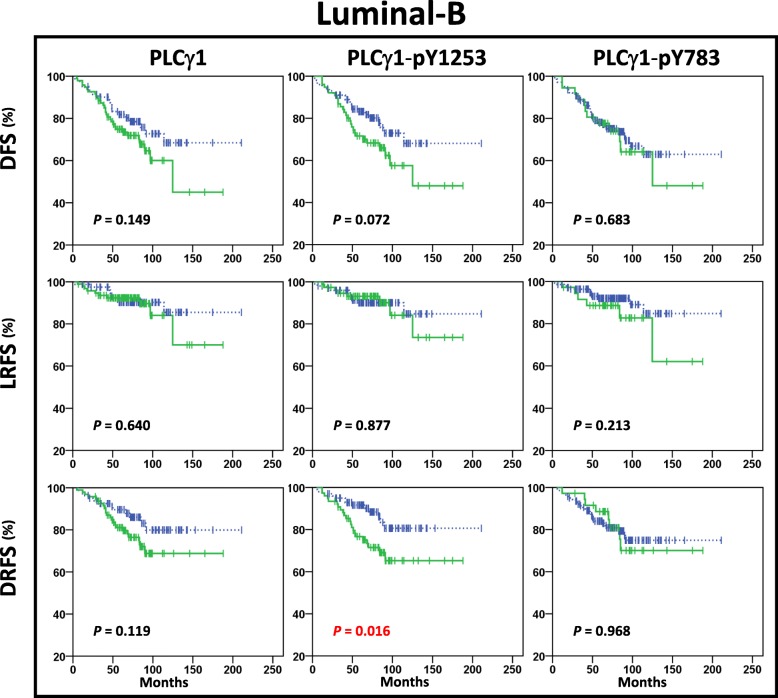
Kaplan-Meier plots in Luminal-B subtype. Kaplan-Meier estimates of DFS, LRFS and DRFS in patients with Luminal-B tumours (*n* = 176), according to high (solid green lines) and low (dashed blue lines) expression of PLCγ1, PLCγ1-pY1253 and PLCγ1-pY783. In this cohort, the patients showed distant relapse in 15% (12/81) PLCγ1^Low^ and 24% (23/95) PLCγ1^High^, in 13% (13/100) PLCγ1-pY1253^Low^ and 29% (22/76) PLCγ1-pY1253^High^, and in 19% (27/140) PLCγ1-pY783^Low^ and 22% (8/36) PLCγ1-pY783^High^

According to the Kaplan-Meier analysis, high expression of PLCγ1 or its activated forms was significantly correlated with lower DFS rates in patients with Luminal-A tumours (Fig. 1), but not in patients with Luminal-B tumours (Fig. 3), or HER2 positive (Additional file 2: Figure S3) or Triple Negative tumours (Additional file 2: Figure S4). Significantly higher rates of distant relapse were seen for Luminal-A pY1253-PLCγ1^High^ (*P* = 0.029) and pY783-PLCγ1^High^ (*P* < 0.001) tumours (Fig. 2). In Luminal-B tumours, those that were pY1253-PLCγ1^High^ (*P* = 0.016), but not those that were pY783-PLCγ1^High^ (*P* = 0.968) showed significantly increased risk of distant relapse (Fig. 3). Multivariate analysis showed that high expression of PLCγ1-pY1253 was an independent prognostic marker for DRFS in Luminal-B tumours (HR, 2.3; 95% CI, 1.2–4.6; *P* = 0.017), while only high expression of PLCγ1-pY783 was correlated with significantly higher risk of distant relapse in patients with Luminal-A tumours (HR, 7.4; 95% CI, 2.3–24.3; *P* = 0.001) (Additional file 1: Table S2).

Kaplan-Meier and multivariate analyses of PLCγ1 transcript expression in tumours from the 1,881-sample breast cancer dataset (GOBO; Gene expression-based Outcome for Breast cancer Online; http://co.bmc.lu.se/gobo) [16], which further confirmed the negative prognostic value of high PLCγ1 protein expression in lymph-node-negative Luminal-A tumours (*n* = 184) (*P* < 0.005, *P* = 0.004, respectively) (Additional file 2: Figure S5). Consistent with this, significantly higher risk of metastatic relapse in patients with Luminal-A, lymph-node negative, PLCγ1^High^ tumours (*n* = 546) was also found in the KM-Plotter microarray database (HR, 2.19; 95% CI, 1.23–3.89; *P* = 0.0065) (Additional file 2: Figure S6) [17].

#### Menopausal status

**Fig. 4:**
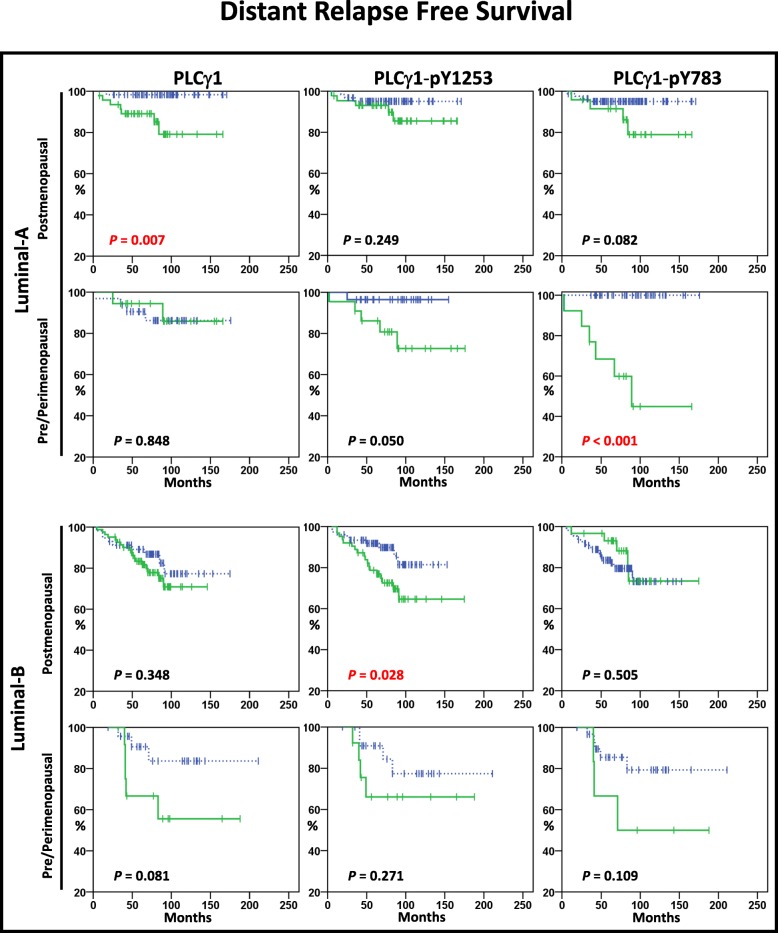
Kaplan-Meier plots in Luminal-A and Luminal-B subtypes. Kaplan-Meier estimates of DRFS in patients with Luminal-A and Luminal-B tumours according to menopausal status. In Luminal-A tumours, the patients with postmenopausal status showed distant relapse in 5% (3/62) PLCγ1-pY1253^Low^ and 11% (5/44) PLCγ1-pY1253^High^, and in 5% (4/82) PLCγ1-pY783^Low^ and 17% (4/24) PLCγ1-pY783^High^, and the patients with premenopausal status showed distant relapse in 4% (1/28) PLCγ1-pY1253^Low^ and 23% (5/22) PLCγ1-pY1253^High^, and in 0% (0/37) PLCγ1-pY783^Low^ and 46% (6/13) PLCγ1-pY783^High^. In Luminal-B tumours, the patients with postmenopausal status showed distant relapse in 12% (9/76) PLCγ1-pY1253^Low^ and 29% (18/63) PLCγ1-pY1253^High^, and in 20% (22/109) PLCγ1-pY783^Low^ and 17% (5/30) PLCγ1-pY783^High^, and the patients with premenopausal status showed distant relapse in 17% (4/24) PLCγ1-pY1253^Low^ and 31% (4/13) PLCγ1-pY1253^High^, and in 16% (5/31) PLCγ1-pY783^Low^ and 50% (3/6) PLCγ1-pY783^High^

Using Kaplan-Meier plots, we also examined the distant recurrence rates associated with PLCγ1-pY1253 and PLCγ1-pY783 expression in luminal tumours with patients clustered according to menopausal status. We observed that pY1253-PLCγ1^High^ expression was significantly associated with lower DRFS rate in postmenopausal patients with Luminal-B tumours (*P* = 0.028), while pY783-PLCγ1^High^ expression was significantly correlated with increased risk of distant relapse in those patients with Luminal-A cancers and pre/perimenopausal status (*P* < 0.001) (Fig. 4).

Multivariate analyses confirmed that pY1253-PLCγ1^High^ was a significant independent prognostic factor for postmenopausal Luminal-B cancers (HR, 2.4: 95% CI, 1.1–5.3; *P* = 0.034), while over-expression of PLCγ1-pY783 represented a significant and strong risk factor for pre/perimenopausal patients with Luminal-A tumours (HR, 20.1: 95% CI, 2.2–178.4; *P* = 0.003) (Table 2, Additional file 1: Table S3).

**Table 2:** Risk of distant relapse according to PLCγ1-pY1253 and PLCγ1-pY783 expression in Luminal-A and Luminal-B tumour subtypes depends on menopausal status. Multivariate analysis

Menopause status	Tumour subtype	PLCγ1-pY1253			PLCγ1-pY783		
		HR^a^	95% CI^b^	*P*	HR^a^	95% CI^b^	*P*
Pre/Perimenopausal	Luminal A	7.5	0.8–71.8	0.079	20.1	2.2–178.4	**0.003**
	Luminal B	2.5	0.6–10.3	0.195	2.9	0.7–12.2	0.149
Postmenopausal	Luminal A	2.2	0.5–9.4	0.274	2.6	0.7–10.7	0.172
	Luminal B	2.4	1.1–5.3	**0.034**	1.2	0.5–3.2	0.717

^a^*HR* Hazard ratio (high versus low PLCγ1-pY expression)

^b^*CI* Confidence interval; statistically significant *p*-values are formatted in bold

**Fig. 5:**
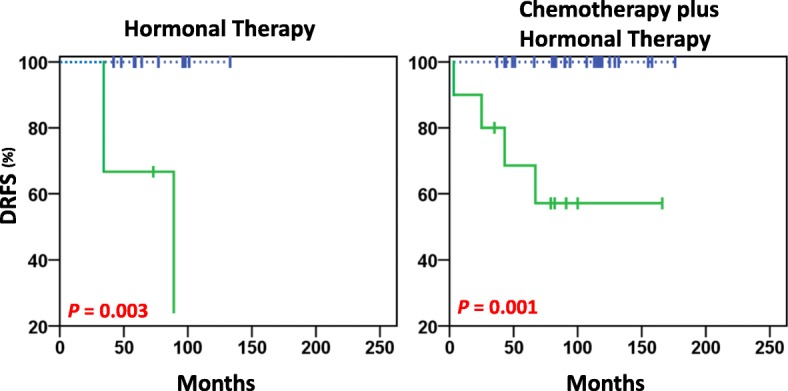
DRFS estimates in Luminal-A premenopausal patients treated with hormonal therapy alone (left) and chemotherapy plus hormonal therapy (right). In this cohort, a distant relapse occurred in 0% (0/12) PLCγ1-pY783^Low^ and 66% (2/3) PLCγ1-pY783^High^ of patients treated with hormonal therapy (*n* = 15), and in 0% (0/23) PLCγ1-pY783^Low^ and 40% (4/10) PLCγ1-pY783^High^ of patients treated with chemotherapy plus hormonal therapy (*n* = 33). The solid green line and dashed blue line represent high and low expression of PLCγ1-pY783, respectively

Significant negative prognostic value was seen for pY783-PLCγ1^High^ for women with Luminal-A tumours who were pre/perimenopausal and were treated with hormonal therapy, as well as those treated with chemotherapy plus hormonal therapy (*P* = 0.003, *P* = 0.001, respectively; Fig. 5). The two patients treated with radiotherapy alone were pY783-PLCγ1^Low^, and they did not show distant metastasis events.

## Discussion

The identification of criteria for accurate prognostication of disease relapse is crucial for the selection of patient candidates for adjuvant therapy. Recognizing patients with high recurrence risk can potentially enhance their treatment outcomes, with the adoption of more aggressive treatments from an earlier stage of the disease, which might ultimately offer better overall survival. Conversely, low-risk patients can undergo less aggressive therapy, and therefore they can enjoy a better quality of life.

PLCγ1 is activated through its phosphorylation by tyrosine kinases in the cell. The major tyrosine kinases that have been shown to activate PLCγ1 in the cell belong to the growth factor receptor superfamily [11], which include the activated receptors for human epidermal growth factor (i.e., HER1/2), fibroblast growth factor, vascular endothelial growth factor, platelet-derived growth factor, hepatocyte growth factor and insulin-like growth factor. Thus, these tyrosine kinase receptors can phosphorylate PLCγ1 on its three tyrosine residues: Y771 and Y783 located between the X and Y catalytic domains, and Y1253 located near the COOH-terminus domain. Upon phosphorylation, PLCγ1 shows increased enzymatic activity, whereby the phosphorylation at Y783 has been described as required for PLCγ1 activation in vitro and in vivo [18–20].

Although the phosphoinositide cycle operates classically at the plasma membrane level, a phosphoinositide cycle operates also within the nucleus [21]. We previously observed a selective nuclear positivity for PLCγ1-pY1253 and PLCγ1-pY783 in patients with early breast cancer, indicating that the nuclear signalling of these activated forms of PLCγ1 may have a specific tumorigenic role [14]. The nuclear PLCγ1 can contribute to mammary carcinogenesis through the modulation of key pathways, including the phosphoinositide 3-kinase (PI3K) nuclear activation [22], and by regulating the expression of cell cycle regulators such as cyclin D1 and cyclin-dependent kinase 4 expression, and the nuclear export of the Cdk inhibitor p27-kip1 [23]. Conversely, the down-regulation of PLCγ1 expression in breast cancer cells results in decreased lung metastasis formation in mice [12]. However, the mechanism/s by which PLCγ1 favours migration and metastatisation remain unclear. PLCγ1 has an essential role in cytoskeletal changes needed for the acquisition of the metastatic phenotype [11, 24], and dephosphorylation of PLCγ1 on residue Y783 inhibits PLCγ1 activation, thus blocking PLCγ1-activated rearrangement of the cytoskeleton, and cell migration [25]. PLCγ1 can contribute to metastatisation by direct [26] or indirect [12, 26] activation of RAC1, thus inducing migration-supporting cellular structures, such as lamellipodia and filopodia.

In the present study, we have shown that the prognostic role of activated PLCγ1 expression is limited to ER-positive, Luminal breast tumours. Indeed, by using validated antibodies for immunohistochemistry on paraffin sections, different PLCγ1 phosphorylation sites were associated with different prognosis for the Luminal-A and -B molecular subtypes. Of note, pY1253-PLCγ1^High^, but not pY783-PLCγ1^High^, was a significant independent prognostic factor for postmenopausal patients with Luminal B cancers (HR, 2.4). On the other hand, this was reversed in the hormonal pre/perimenopausal setting, where pY783-PLCγ1^High^, but not pY1253-PLCγ1^High^, was a particularly strong and significant risk factor for metastatic relapse (HR, 20.1).

Several multigene assays can now be included in clinical practice, such as the Oncotype DX, Prosigna and MammaPrint assays. Compared to the use of standard prognostic criteria, these multigene assays can provide some improvements in the recognition of patients with early stage ER-positive, HER2-negative breast cancer that will be at risk of recurrence. Indeed, at present, randomised controlled trials [27] are ongoing to prospectively validate their clinical usefulness. The future of diagnostic/ prognostic testing in ER-positive breast cancer is likely to rely on devising and reliably deploying assays that can predict the benefits of additional therapies, including newer targeted therapies. As no particular technology holds the key, immunohistochemistry remains a well settled, widely diffuse, and low-cost technique, and so it can have a role in the choice of adequate treatment [28].

Although the relationship between oestrogen stimulation and PLCγ expression has been explored in depth, recent data [29] have indicate a role for PLCγ1 in the proliferation of ER-positive tumour cells. Cells must increase chaperone levels to fold and sort proteins required for ERα-dependent cell proliferation. The unfolded protein response (UPR), which is an endoplasmic reticulum stress sensor, controls protein folding homeostasis. The UPR is overexpressed in several tumours where an early, pathological, activation of UPR occurs before the accumulation of unfolded proteins. In ERα-positive breast and ovary cancer cells, 17β-oestradiol induces rapid anticipatory activation of the UPR that is strictly PLCγ1 dependent. ER-positive breast cancers demonstrate elevated expression of a UPR gene signature that is also a prognostic marker associated to high risk of relapse and poor survival, and also resistance to tamoxifen therapy. Therefore, this PLCγ1-dependent anticipatory activation of the UPR defines a new role for oestrogens that can create a supportive environment for cancer cell proliferation and resistance to therapy, and might represent a new target in breast cancer [30]. Considering this aspect, it has also been reported that PLCγ1 activation downstream of FGFR-3 signalling is a critical event in the control of MAPK and PI3K activation, which can induce resistance to tamoxifen treatment [31].

## Conclusions

Although the mechanisms involved remain to be defined, the activation of PLCγ1 as assessed by immunohistochemistry is a strong prognostic factor that can discriminate between high-risk and low-risk patients with hormone-receptor-positive early breast cancers. PLCγ1 might thus serve as a new target especially for treatment of Luminal-A pre/perimenopausal patients with T1-T2, N0 disease.

## Additional files

Additional file 1: **Table S1.** Multivariate analyses of PLCγ1, PLCγ1-pY1253 and PLCγ1-pY783 expression in all cases (*n* = 414). **Table S2.** Multivariate analyses of PLCγ1-pY1253, pY783 and PLCγ1 expression in Luminal-A and Luminal-B subtypes. **Table S3.** PLCγ1-pY1253 and PLCγ1-pY783 expression in Luminal-A (LA) and Luminal-B (LB) subtypes according to menopausal status: multivariate analyses. (DOCX 49 kb)

Additional file 2: **Figure S1.** Immunoreactivity for PLCγ1, PLCγ1-pY1253 and PLCγ1-pY783 in wild-type (wt) and down-regulated (si) MDA-MB-231 breast cancer cells. **Figure S2.** All patients (*n* = 414): Kaplan-Meier estimates of DFS, LRFS, and DRFS according to high (solid green lines) and low (dashed blue lines) expression of PLCγ1, PLCγ1-pY1253 and PLCγ1-pY783; **Figure S3.** Patients with HER2 positive breast cancer subtype (*n* = 27): Kaplan-Meier estimates of DFS, LRFS, and DRFS according to high (solid green lines) and low (dashed blue lines) expression of PLCγ1, PLCγ1-pY1253 and PLCγ1-pY783. **Figure S4.** Patients with Triple Negative breast cancer subtype (*n* = 55): Kaplan-Meier estimates of DFS, LRFS, and DRFS according to high (solid green lines) and low (dashed blue lines) expression of PLCγ1, PLCγ1-pY1253 and PLCγ1-pY783. **Figure S5.** GOBO (Gene expression-based Outcome for Breast cancer Online) database (http://co.bmc.lu.se/gobo): Kaplan-Meier plot of DFS (A) and multivariate (B) analyses of *PLCG1* transcript expression in lymph-node-negative HU-Luminal A tumours (*n* = 184). Red and grey lines represent tumours expressing high and low *PLCG1* mRNA levels, respectively. **Figure S6.** KM-Plotter microarray database (http://kmplot.com/analysis/index.php?p=service&cancer=breast): Kaplan-Meier plot of distant metastasis-free survival (DMFS) of *PLCG1* transcript expression in Luminal-A lymph-node negative breast cancer patients (*n* = 546). Red and black lines represent tumours expressing high and low *PLCG1* mRNA levels, respectively. (PPTX 1483 kb)

## Data Availability

The data that support the findings of this study are not available publicly. However, the data are available from the corresponding author upon reasonable request.

## Abbreviations

CI: Confidence interval
DFS: Disease-free survival
DRFS: Distant relapse-free survival
ER: Oestrogen receptor
HR: Hazard ratio
LRFS: Local relapse-free survival
PgR: Progesterone receptor
PLCγ1: Phospholipase Cγ1
UPR: Unfolded protein response
