# Supplementary material for: PLC-gamma-1 phosphorylation status is prognostic of metastatic risk in patients with early-stage Luminal-A and -B breast cancer subtypes
Source: BMC Cancer. 2019 Jul 30;19:747. doi: 10.1186/s12885-019-5949-x (PMC6668079; doi:10.1186/s12885-019-5949-x)
Supplement: Supplementary file 1 — Table S1. Multivariate analyses of PLCγ1, PLCγ1-pY1253 and PLCγ1-pY783 expression in all cases (n = 414). Table S2. Multivariate analyses of PLCγ1-pY1253, pY783 and PLCγ1 expression in Luminal-A and Luminal-B subtypes. Table S3. PLCγ1-pY1253 and PLCγ1-pY783 expression in Luminal-A (LA) and Luminal-B (LB) subtypes according to menopausal status: multivariate analyses. (DOCX 49 kb) [file 12885_2019_5949_MOESM1_ESM.docx]

**Table S1 .**Multivariate analyses of PLCγ1, PLCγ1-pY1253, and PLCγ1-pY783 expression in all cases (n = 414)

|  | **PLCγ1** | | | |  | **PLCγ1-pY1253** | | | |  | **PLCγ1-pY783** | | | |
| --- | --- | --- | --- | --- | --- | --- | --- | --- | --- | --- | --- | --- | --- | --- |
|  |  | **HR** | **95% CI** | **P** |  |  | **HR** | **95% CI** | **P** |  | **Variable** | **HR** | **95% CI** | **P** |
|  |  |  |  |  |  |  |  |  |  |  |  |  |  |  |
| **DFS** | Tumor size, cm (≤ 2 *vs* > 2) | 1.1 | 0.8-1.7 | 0.564 |  | Tumor size, cm (≤ 2 *vs* > 2) | 1.2 | 0.8-1.8 | 0.387 |  | Tumor size, cm (≤ 2 *vs* > 2) | 1.2 | 0.8-1.7 | 0.464 |
|  | **Tumor grade (2-3 *vs* 1)** | **2.3** | **1.0-5.0** | **0.040** |  | **Tumor grade (2-3 *vs* 1)** | **2.4** | **1.1-5.3** | **0.026** |  | **Tumor grade (2-3 *vs* 1)** | **2.5** | **1.1-5.4** | **0.024** |
|  | ER (negative *vs* positive) | 1.1 | 0.7-1.9 | 0.695 |  | ER (negative *vs* positive) | 1.1 | 0.6-1.8 | 0.841 |  | ER (negative *vs* positive) | 1.0 | 0.6-1.7 | 0.923 |
|  | PR (negative *vs* positive) | 1.4 | 0.9-2.2 | 0.135 |  | PR (negative *vs* positive) | 1.5 | 0.9-2.3 | 0.091 |  | PR (negative *vs* positive) | 1.5 | 0.9-2.3 | 0.094 |
|  | Ki-67 (high *vs* low) | 1.5 | 0.9-2.2 | 0.080 |  | Ki-67 (high *vs* low) | 1.5 | 0.9-2.3 | 0.061 |  | Ki-67 (high *vs* low) | 1.5 | 0.9-2.3 | 0.079 |
|  | HER-2 (positive *vs* negative) | 1.2 | 0.7-1.9 | 0.453 |  | HER-2 (positive *vs* negative) | 1.2 | 0.8-2.0 | 0.387 |  | HER-2 (positive *vs* negative) | 1.3 | 0.8-2.1 | 0.274 |
|  | **PLCγ1 (high *vs* low)** | **1.5** | **1.0-2.3** | **0.029** |  | **PLCγ1-pY1253 (high *vs* low)** | **1.6** | **1.1-2.3** | **0.024** |  | PLCγ1-pY783 (high *vs* low) | 1.3 | 0.9-2.0 | 0.153 |
|  |  |  |  |  |  |  |  |  |  |  |  |  |  |  |
| **LRFS** | Tumor size, cm (>2 *vs* ≤ 2) | 1.1 | 0.6-2.1 | 0.683 |  | Tumor size, cm (>2 *vs* ≤ 2) | 1.1 | 0.6-2.1 | 0.690 |  | Tumor size, cm (> 2 *vs* ≤ 2) | 1.1 | 0.6-2.1 | 0.678 |
|  | Tumor grade (2-3 *vs* 1) | 1.5 | 0.6-3.8 | 0.440 |  | Tumor grade (2-3 *vs* 1) | 1.5 | 0.6-3.9 | 0.419 |  | Tumor grade (2-3 *vs* 1) | 1.5 | 0.6-3.9 | 0.415 |
|  | ER (positive *vs* negative) | 1.0 | 0.5-2.3 | 0.908 |  | ER (positive *vs* negative) | 1.1 | 0.5-2.4 | 0.863 |  | ER (positive *vs* negative) | 1.1 | 0.5-2.4 | 0.840 |
|  | PR (negative *vs* positive) | 1.8 | 0.9-3.4 | 0.094 |  | PR (negative *vs* positive) | 1.8 | 0.9-3.4 | 0.078 |  | PR (negative *vs* positive) | 1.8 | 0.9-3.5 | 0.077 |
|  | Ki-67 (high *vs* low) | 1.2 | 0.6-2.2 | 0.665 |  | Ki-67 (high *vs* low) | 1.2 | 0.6-2.2 | 0.661 |  | Ki-67 (high *vs* low) | 1.2 | 0.6-2.2 | 0.662 |
|  | HER-2 (negative *vs* positive) | 1.3 | 0.6-3.1 | 0.481 |  | HER-2 (negative *vs* positive) | 1.3 | 0.6-3.0 | 0.496 |  | HER-2 (negative *vs* positive) | 1.3 | 0.6-3.0 | 0.492 |
|  | PLCγ1 (high *vs* low) | 1.1 | 0.6-2.0 | 0.758 |  | PLCγ1-pY1253 (high *vs* low) | 1.0 | 0.6-1.8 | 0.935 |  | PLCγ1-pY783 (low *vs* high) | 1.0 | 0.6-1.8 | 0.893 |
|  |  |  |  |  |  |  |  |  |  |  |  |  |  |  |
| **DRFS** | Tumor size, cm (≤ 2 *vs* > 2) | 1.3 | 0.8-2.2 | 0.347 |  | Tumor size, cm (≤ 2 *vs* > 2) | 1.4 | 0.8-2.4 | 0.201 |  | Tumor size, cm (≤ 2 *vs* > 2) | 1.4 | 0.8-2.3 | 0.260 |
|  | **Tumor grade (2-3 *vs* 1)** | **4.6** | **1.1-19.1** | **0.037** |  | **Tumor grade (2-3 *vs* 1)** | **5.1** | **1.2-21.1** | **0.025** |  | **Tumor grade (2-3 *vs* 1)** | **5.3** | **1.3-22.1** | **0.022** |
|  | ER (negative *vs* positive) | 1.0 | 0.5-2.0 | 0.925 |  | ER (positive *vs* negative) | 1.1 | 0.6-2.2 | 0.766 |  | ER (positive *vs* negative) | 1.2 | 0.6-2.3 | 0.686 |
|  | PR (negative *vs* positive) | 1.3 | 0.7-2.3 | 0.406 |  | PR (negative *vs* positive) | 1.3 | 0.8-2.4 | 0.323 |  | PR (negative *vs* positive) | 1.4 | 0.8-2.4 | 0.307 |
|  | Ki-67 (high *vs* low) | 1.6 | 0.9-2.8 | 0.088 |  | Ki-67 (high *vs* low) | 1.8 | 0.9-3.1 | 0.052 |  | Ki-67 (high *vs* low) | 1.6 | 0.9-2.9 | 0.083 |
|  | HER-2 (positive *vs* negative) | 1.5 | 0.8-2.6 | 0.201 |  | HER-2 (positive *vs* negative) | 1.5 | 0.8-2.7 | 0.183 |  | HER-2 (positive *vs* negative) | 1.7 | 0.9-2.9 | 0.087 |
|  | **PLCγ1 (high *vs* low)** | **2.1** | **1.3-3.6** | **0.005** |  | **PLCγ1-pY1253 (high *vs* low)** | **2.3** | **1.4-3.7** | **0.001** |  | **PLCγ1-pY783 (high *vs* low)** | **1.7** | **1.0-2.7** | **0.049** |
|  |  |  |  |  |  |  |  |  |  |  |  |  |  |  |

**Table S2 .** Multivariate analyses of PLCγ1-pY1253, pY783 and PLCγ1 expression in Luminal-A and Luminal-B subtypes

|  | **LUMINAL A** | | | |  | **LUMINAL B** | | | |
| --- | --- | --- | --- | --- | --- | --- | --- | --- | --- |
|  |  | **HR** | **95% CI** | **P** |  |  | **HR** | **95% CI** | **P** |
|  | **PLCγ1** |  |  |  |  | **PLCγ1** |  |  |  |
| **DFS** | Tumor size, cm (≤ 2 *vs* > 2) | 1.5 | 0.6-3.5 | 0.363 |  | Tumor size, cm (≤ 2 *vs* > 2) | 1.0 | 0.6-1.9 | 0.894 |
|  | Tumor grade (2-3 *vs* 1) | 2.3 | 0.8-6.7 | 0.127 |  | Tumor grade (2-3 *vs* 1) | 3.8 | 0.9-16.0 | 0.067 |
|  | **PLCγ1 (high *vs* low)** | **2.2** | **1.1-4.5** | **0.031** |  | PLCγ1 (high *vs* low) | 1.3 | 0.8-2.4 | 0.316 |
|  |  |  |  |  |  |  |  |  |  |
| **LRFS** | Tumor size, cm (≤ 2 *vs* > 2) | 1.0 | 0.4-2.9 | 0.979 |  | Tumor size, cm (> 2 *vs* ≤ 2) | 1.1 | 0.4-3.0 | 0.776 |
|  | Tumor grade (2-3 *vs* 1) | 1.5 | 0.4-5.3 | 0.539 |  | Tumor grade (2-3 *vs* 1) | 2.6 | 0.3-20.0 | 0.368 |
|  | PLCγ1 (high *vs* low) | 2.1 | 0.8-5.3 | 0.129 |  | PLCγ1 (low *vs* high) | 1.1 | 0.4-2.9 | 0.807 |
|  |  |  |  |  |  |  |  |  |  |
| **DRFS** | Tumor size, cm (≤ 2 *vs* > 2) | 1.6 | 0.4-5.9 | 0.459 |  | Tumor size, cm (≤ 2 *vs* > 2) | 1.2 | 0.6-2.5 | 0.576 |
|  | Tumor grade (2-3 *vs* 1) | 4.4 | 0.6-34.4 | 0.155 |  | Tumor grade (2-3 *vs* 1) | 5.4 | 0.7-40.1 | 0.099 |
|  | PLCγ1 (high *vs* low) | 2.6 | 0.9-7.6 | 0.093 |  | PLCγ1 (high *vs* low) | 1.5 | 0.7-3.1 | 0.250 |
|  |  |  |  |  |  |  |  |  |  |
|  | **pY1253** |  |  |  |  | **pY1253** |  |  |  |
| **DFS** | Tumor size, cm (≤ 2 *vs* > 2) | 1.7 | 0.7-3.9 | 0.241 |  | Tumor size, cm (≤ 2 *vs* > 2) | 1.1 | 0.6-2.0 | 0.728 |
|  | Tumor grade (2-3 *vs* 1) | 2.2 | 0.8-6.4 | 0.141 |  | **Tumor grade (2-3 *vs* 1)** | **4.2** | **1.0-17.4** | **0.048** |
|  | pY1253 (high *vs* low) | 2.0 | 0.9-4.2 | 0.057 |  | pY1253 (high *vs* low) | 1.7 | 0.9-3.0 | 0.075 |
|  |  |  |  |  |  |  |  |  |  |
| **LRFS** | Tumor size, cm (≤ 2 *vs* > 2) | 1.1 | 0.4-3.2 | 0.866 |  | Tumor size, cm (> 2 *vs* ≤ 2) | 1.1 | 0.4-3.0 | 0.783 |
|  | Tumor grade (2-3 *vs* 1) | 1.5 | 0.4-5.3 | 0.534 |  | Tumor grade (2-3 *vs* 1) | 2.7 | 0.3-20.4 | 0.345 |
|  | pY1253 (high *vs* low) | 1.5 | 0.6-3.9 | 0.400 |  | pY1253 (low *vs* high) | 1.1 | 0.4-2.7 | 0.910 |
|  |  |  |  |  |  |  |  |  |  |
| **DRFS** | Tumor size, cm (≤ 2 *vs* > 2) | 1.9 | 0.5-6.9 | 0.333 |  | Tumor size, cm (≤ 2 *vs* > 2) | 1.4 | 0.7-2.8 | 0.407 |
|  | Tumor grade (2-3 *vs* 1) | 4.1 | 0.5-31.6 | 0.180 |  | Tumor grade (2-3 *vs* 1) | 6.2 | 0.8-45.3 | 0.074 |
|  | pY1253 (high *vs* low) | 3.2 | 0.9-10.3 | 0.050 |  | **pY1253 (high *vs* low)** | **2.3** | **1.2-4.6** | **0.017** |
|  |  |  |  |  |  |  |  |  |  |
|  |  |  |  |  |  |  |  |  |  |
|  | **pY783** |  |  |  |  | **pY783** |  |  |  |
| **DFS** | Tumor size, cm (≤ 2 *vs* > 2) | 1.6 | 0.7-3.8 | 0.277 |  | Tumor size, cm (≤ 2 *vs* > 2) | 1.0 | 0.6-1.9 | 0.905 |
|  | Tumor grade (2-3 *vs* 1) | 1.8 | 0.6-5.3 | 0.293 |  | **Tumor grade (2-3 *vs* 1)** | **4.3** | **1.0-17.9** | **0.045** |
|  | **pY783 (high *vs* low)** | **3.1** | **1.5-6.4** | **0.002** |  | pY783 (high *vs* low) | 1.3 | 0.7-2.4 | 0.502 |
|  |  |  |  |  |  |  |  |  |  |
| **LRFS** | Tumor size, cm (≤ 2 *vs* > 2) | 1.0 | 0.4-3.0 | 0.943 |  | Tumor size, cm (> 2 *vs* ≤ 2) | 1.2 | 0.5-3.1 | 0.732 |
|  | Tumor grade (2-3 *vs* 1) | 1.4 | 0.4-5.1 | 0.599 |  | Tumor grade (2-3 *vs* 1) | 2.9 | 0.4-22.4 | 0.306 |
|  | pY783 (high *vs* low) | 1.5 | 0.5-4.0 | 0.443 |  | pY783 (high *vs* low) | 2.0 | 0.7-5.3 | 0.170 |
|  |  |  |  |  |  |  |  |  |  |
| **DRFS** | Tumor size, cm (≤ 2 *vs* > 2) | 1.9 | 0.5-6.9 | 0.333 |  | Tumor size, cm (≤ 2 *vs* > 2) | 1.2 | 0.6-2.5 | 0.564 |
|  | Tumor grade (2-3 *vs* 1) | 2.6 | 0.3-21.2 | 0.366 |  | Tumor grade (2-3 *vs* 1) | 6.2 | 0.8-45.9 | 0.073 |
|  | **pY783 (high *vs* low)** | **7.4** | **2.3-24.3** | **0.001** |  | pY783 (high *vs* low) | 1.1 | 0.5-2.5 | 0.791 |
|  |  |  |  |  |  |  |  |  |  |

**Table S3.** PLCγ1-pY1253 and PLCγ1-pY783 expressions in Luminal-A (LA) and Luminal-B (LB) subtypes according to menopausal status: multivariate analyses.

|  |  | **Postmenopausal Luminal A** | | | | **Postmenopausal Luminal B** | | | | **Pre/Perimenopausal Luminal A** | | | | **Pre/Perimenopausal Luminal B** | | | |
| --- | --- | --- | --- | --- | --- | --- | --- | --- | --- | --- | --- | --- | --- | --- | --- | --- | --- |
|  |  | Variable | HR | 95% CI | P | Variable | HR | 95% CI | P | Variable | HR | 95% CI | P | Variable | HR | 95% CI | P |
|  |  |  |  |  |  |  |  |  |  |  |  |  |  |  |  |  |  |
| **PLCγ1-pY1253** | **DFS** | T, cm (≤2 *vs* >2) | 1.1 | 0.4-3.2 | 0.815 | T, cm (>2 *vs* ≤2) | 1.1 | 0.6-2.1 | 0.726 | T, cm (≤2 *vs* >2) | 4.6 | 0.6-36.2 | 0.146 | T, cm (≤ 2 *vs* >2) | 4.9 | 0.6-39.6 | 0.139 |
|  |  | Grade (2-3 *vs* 1) | 4.6 | 0.6-35.0 | 0.145 | Grade (2-3 *vs* 1) | 6.4 | 0.9-47.0 | 0.068 | Grade (2-3 *vs*1) | 1.2 | 0.3-4.6 | 0.801 | Grade (2-3 *vs* 1) | 2.1 | 0.3-16.6 | 0.487 |
|  |  | pY1253 (high *vs* low) | 2.2 | 0.8-6.2 | 0.124 | pY1253 (high *vs* low) | 1.7 | 0.9-3.2 | 0.113 | pY1253 (high *vs* low) | 2.2 | 0.7-6.7 | 0.155 | pY1253 (high *vs* low) | 1.8 | 0.5-6.3 | 0.390 |
|  | **LRFS** | T, cm (>2 *vs* ≤2) | 1.8 | 0.4-7.6 | 0.397 | T, cm (>2 *vs* ≤2) | 1.3 | 0.5-3.6 | 0.618 | T, cm (≤2 *vs* >2) | 1.9 | 0.2-16.0 | 0.558 | T,cm (≤ 2 *vs* > 2) | 2.1 | 0.4-1.9 | 0.615 |
|  |  | Grade (2-3 *vs* 1) | 2.2 | 0.3-18.0 | 0.471 | Grade (2-3 *vs* 1) | 2.3 | 0.3-17.6 | 0.437 | Grade (2-3 *vs* 1) | 1.1 | 0.2-6.1 | 0.906 | Grade (2-3 *vs* 1) | 1.4 | 0.3-7.0 | 0.691 |
|  |  | pY1253 (high *vs* low) | 2.6 | 0.6-10.4 | 0.184 | pY1253 (high *vs* low) | 1.1 | 0.4-3.1 | 0.789 | pY1253 (high *vs* low) | 1.0 | 0.3-4.2 | 0.967 | pY1253 (low *vs* high) | 5.2 | 0.3-4.0 | 0.564 |
|  | **DRFS** | T, cm (≤2 *vs* >2) | 1.0 | 0.2-4.3 | 0.978 | T, cm (≤2 *vs* >2) | 1.1 | 0.5-2.4 | 0.784 | T, cm (≤2 *vs* >2) | 1.1 | 0.1-10.4 | 0.984 | T, cm (≤ 2 *vs* >2) | 3.8 | 0.5-31.8 | 0.221 |
|  |  | Grade (2-3 *vs* 1) | 1.0 | 0.3-4.5 | 0.980 | Grade (2-3 *vs* 1) | 1.5 | 0.3-58.1 | 0.970 | Grade (2-3 *vs* 1) | 1.2 | 0.1-11.5 | 0.871 | Grade (2-3 *vs* 1) | 1.5 | 0.2-12.4 | 0.707 |
|  |  | pY1253 (high *vs* low) | 2.2 | 0.5-9.4 | 0.274 | **pY1253 (high *vs* low)** | **2.4** | **1.1-5.3** | **0.034** | pY1253 (high *vs* low) | 7.5 | 0.8-71.8 | 0.079 | pY1253 (high *vs* low) | 2.5 | 0.6-10.3 | 0.195 |
|  |  |  |  |  |  |  |  |  |  |  |  |  |  |  |  |  |  |
| **PLCγ1-pY783** | **DFS** | T, cm (>2 *vs* ≤2) | 1.2 | 0.4-3.5 | 0.711 | T, cm (>2 *vs* ≤2) | 1.2 | 0.6-2.2 | 0.621 | T, cm (≤2 *vs* >2) | 6.4 | 0.8-51.2 | 0.082 | T, cm (≤ 2 *vs* >2) | 4.1 | 0.5-32.8 | 0.181 |
|  |  | Grade (2-3 *vs* 1) | 4.0 | 0.5-30.9 | 0.188 | Grade (2-3 *vs* 1) | 6.5 | 0.9-47.7 | 0.067 | Grade (1 *vs* 2-3) | 2.2 | 0.4-10.8 | 0.334 | Grade (2-3 *vs* 1) | 2.2 | 0.3-17.5 | 0.459 |
|  |  | pY783 (high *vs* low) | 1.6 | 0.6-4.5 | 0.355 | pY783 (high *vs* low) | 1.1 | 0.5-2.3 | 0.816 | **pY783 (high *vs* low)** | **14.4** | **3.3-62.1** | **0.000** | pY783 (high *vs* low) | 2.1 | 0.5-8.2 | 0.287 |
|  | **LRFS** | T, cm (>2 *vs* ≤2) | 2.0 | 0.5-8.3 | 0.330 | T, cm (>2 *vs* ≤2) | 1.4 | 0.5-3.9 | 0.508 | T, cm (≤2 *vs* >2) | 1.9 | 0.2-15.7 | 0.555 | T, cm (≤ 2 *vs* >2) | 3.0 | 0.3-2.1 | 0.585 |
|  |  | Grade (2-3 *vs* 1) | 1.9 | 0.2-15.7 | 0.573 | Grade (2-3 *vs* 1) | 2.6 | 0.3-20.4 | 0.369 | Grade (2-3 *vs* 1) | 1.1 | 0.2-6.0 | 0.954 | Grade (2-3 *vs* 1) | 1.4 | 0.3-6.9 | 0.694 |
|  |  | pY783 (high *vs* low) | 1.4 | 0.3-5.9 | 0.636 | pY783 (high *vs* low) | 2.2 | 0.8-6.2 | 0.123 | pY783 (high *vs* low) | 1.6 | 0.4-7.0 | 0.539 | pY783 (low *vs* high) | 4.9 | 0.3-3.6 | 0.661 |
|  | **DRFS** | T, cm (>2 *vs* ≤2) | 1.0 | 0.2-4.3 | 0.984 | T, cm (≤2 *vs* >2) | 1.1 | 0.5-2.3 | 0.902 | T, cm (≤2 *vs* >2) | 6.7 | 0.4-22.1 | 0.402 | T, cm (≤ 2 *vs* >2) | 2.9 | 0.4-23.9 | 0.321 |
|  |  | Grade (2-3 *vs* 1) | 1.0 | 0.2-4.5 | 0.971 | Grade (2-3 *vs* 1) | 1.3 | 0.4-56.0 | 0.972 | Grade (2-3 *vs* 1) | 5.9 | 0.5-21.8 | 0.425 | Grade (2-3 *vs* 1) | 1.6 | 0.2-13.2 | 0.664 |
|  |  | pY783 (high *vs* low) | 2.6 | 0.7-10.7 | 0.172 | pY783(low *vs* high) | 1.2 | 0.5-3.2 | 0.717 | **pY783 (high *vs* low)** | **20.1** | **2.2-178.4** | **0.003** | pY783 (high *vs* low) | 2.9 | 0.7-12.2 | 0.149 |
|  |  |  |  |  |  |  |  |  |  |  |  |  |  |  |  |  |  |
