# Supplementary material for: PLC-gamma-1 phosphorylation status is prognostic of metastatic risk in patients with early-stage Luminal-A and -B breast cancer subtypes
Source: BMC Cancer. 2019 Jul 30;19:747. doi: 10.1186/s12885-019-5949-x (PMC6668079; doi:10.1186/s12885-019-5949-x)
Supplement: Supplementary file 2 — Figure S1. Immunoreactivity for PLCγ1, PLCγ1-pY1253 and PLCγ1-pY783 in wild-type (wt) and down-regulated (si) MDA-MB-231 breast cancer cells. Figure S2. All patients (n = 414): Kaplan-Meier estimates of DFS, LRFS, and DRFS according to high (solid green lines) and low (dashed blue lines) expression of PLCγ1, PLCγ1-pY1253 and PLCγ1-pY783; Figure S3. Patients with HER2 positive breast cancer subtype (n = 27): Kaplan-Meier estimates of DFS, LRFS, and DRFS according to high (solid green lines) and low (dashed blue lines) expression of PLCγ1, PLCγ1-pY1253 and PLCγ1-pY783. Figure S4. Patients with Triple Negative breast cancer subtype (n = 55): Kaplan-Meier estimates of DFS, LRFS, and DRFS according to high (solid green lines) and low (dashed blue lines) expression of PLCγ1, PLCγ1-pY1253 and PLCγ1-pY783. Figure S5. GOBO (Gene expression-based Outcome for Breast cancer Online) database (http://co.bmc.lu.se/gobo): Kaplan-Meier plot of DFS (A) and multivariate (B) analyses of PLCG1 transcript expression in lymph-node-negative HU-Luminal A tumours (n = 184). Red and grey lines represent tumours expressing high and low PLCG1 mRNA levels, respectively. Figure S6. KM-Plotter microarray database (http://kmplot.com/analysis/index.php?p=service&cancer=breast): Kaplan-Meier plot of distant metastasis-free survival (DMFS) of PLCG1 transcript expression in Luminal-A lymph-node negative breast cancer patients (n = 546). Red and black lines represent tumours expressing high and low PLCG1 mRNA levels, respectively. (PPTX 1483 kb) [file 12885_2019_5949_MOESM2_ESM.pptx]

## Slide 1
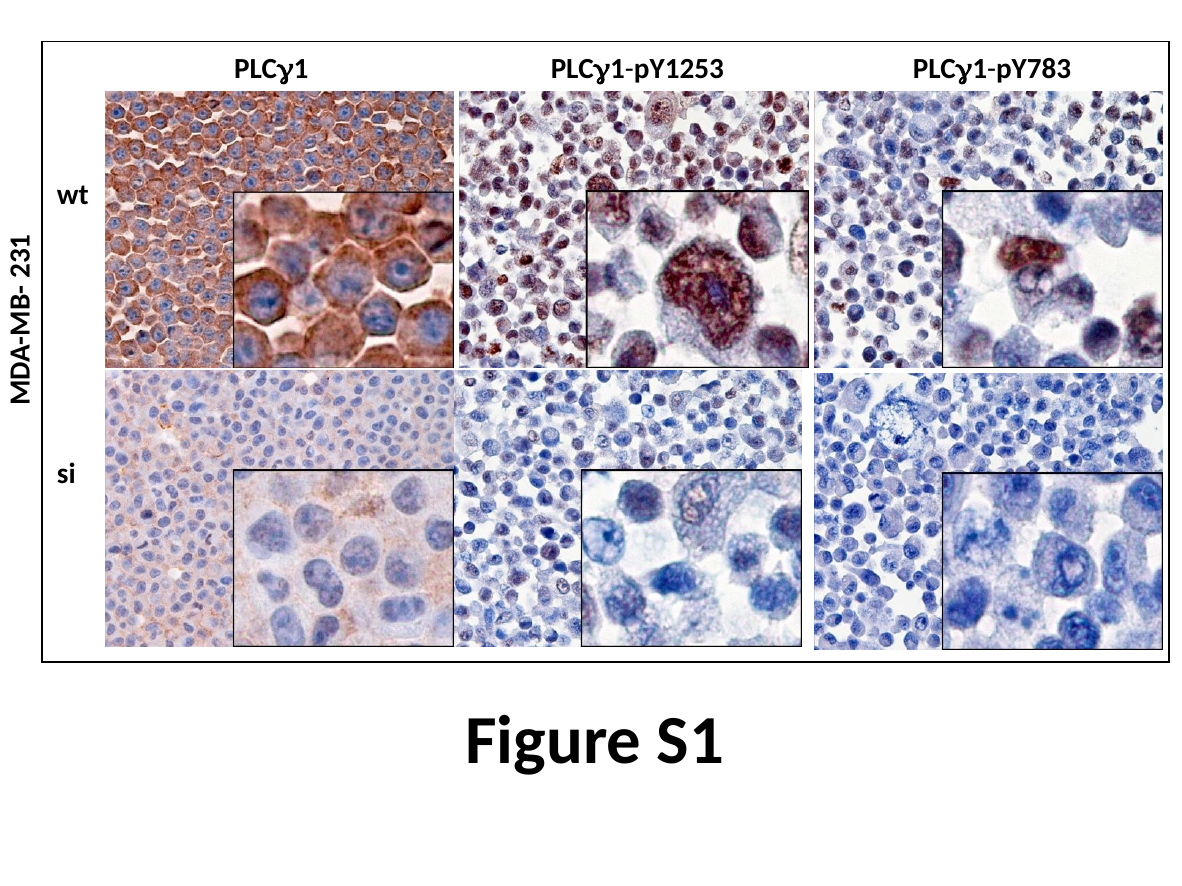

PLCγ1
PLCγ1-pY1253
PLCγ1-pY783
wt
MDA-MB- 231
si
Figure S1

## Slide 2
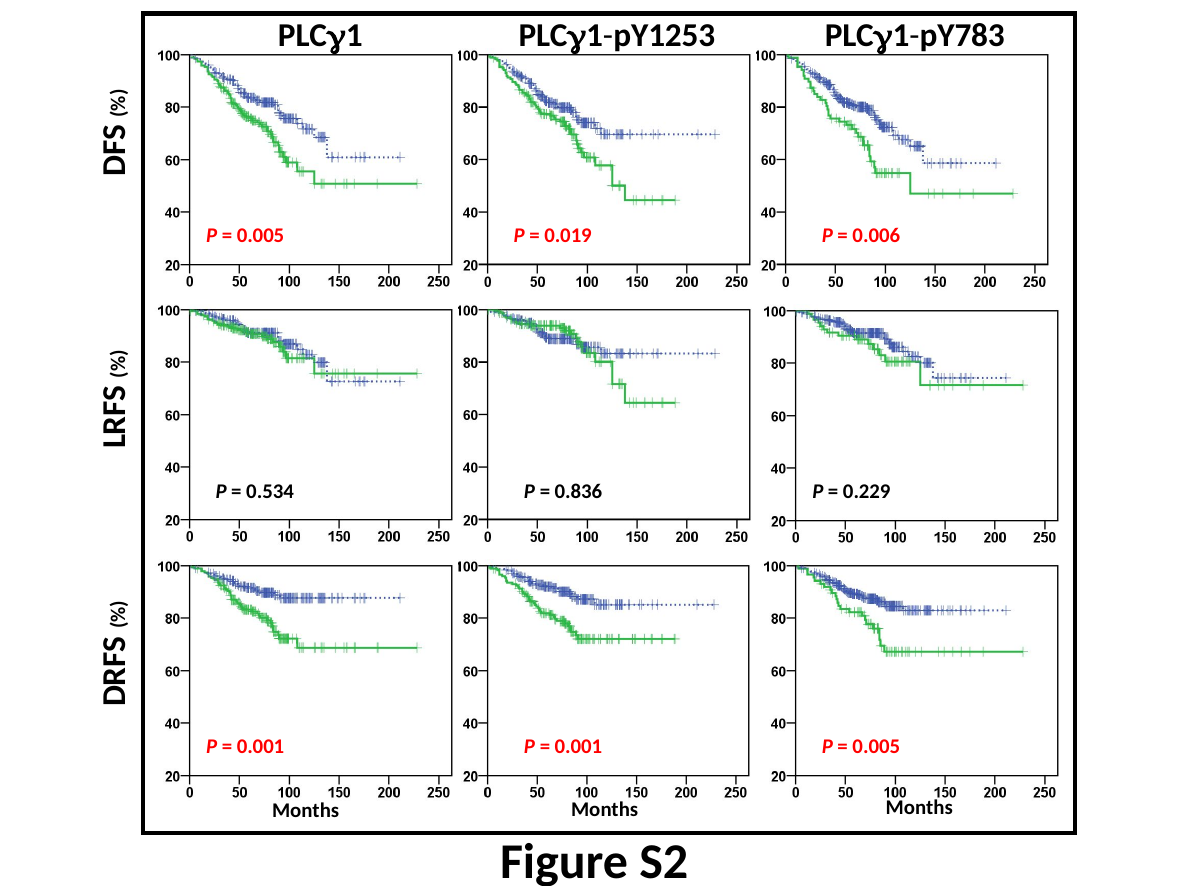

PLCγ1
PLCγ1-pY1253
PLCγ1-pY783
DFS (%)
P = 0.005
P = 0.019
P = 0.006
LRFS (%)
P = 0.534
P = 0.836
P = 0.229
DRFS (%)
P = 0.001
P = 0.001
P = 0.005
Months
Months
Months
Figure S2

## Slide 3
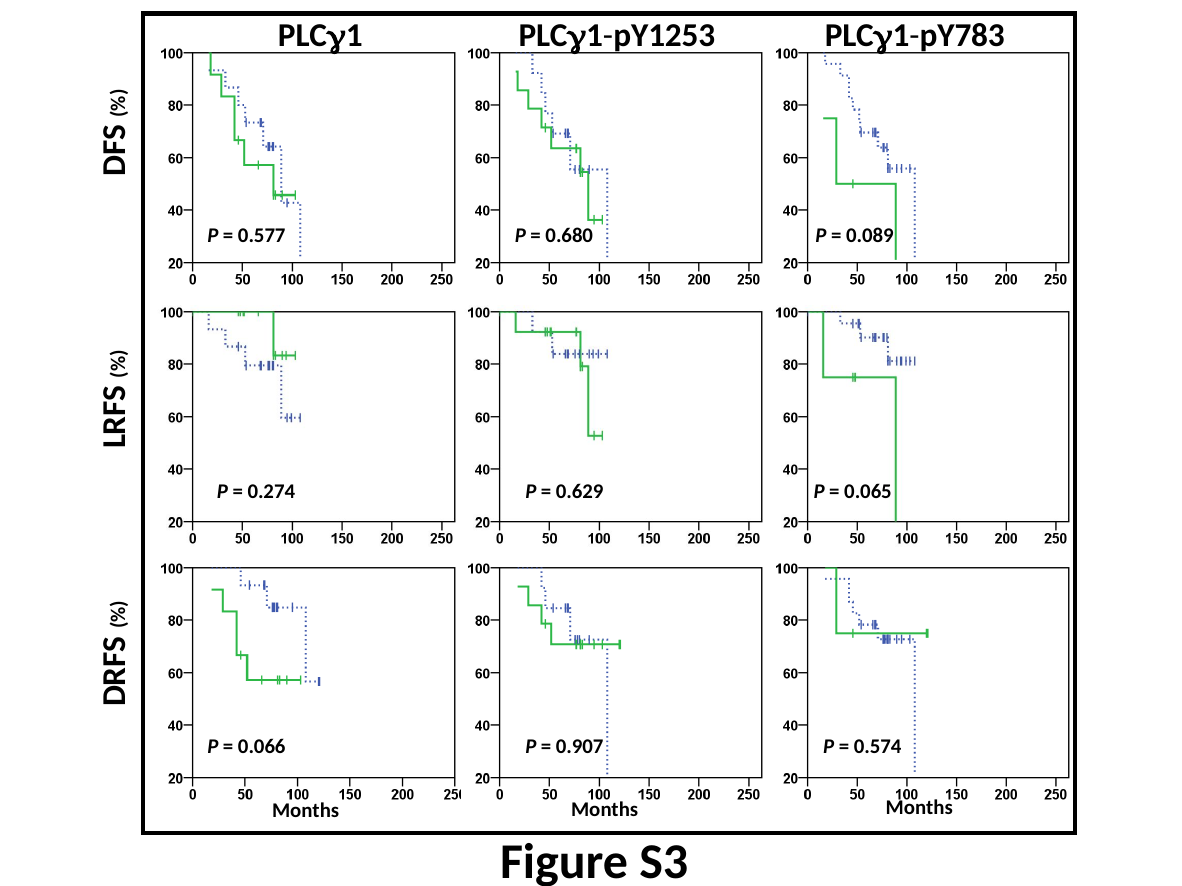

PLCγ1
PLCγ1-pY1253
PLCγ1-pY783
DFS (%)
P = 0.577
P = 0.680
P = 0.089
LRFS (%)
P = 0.274
P = 0.629
P = 0.065
DRFS (%)
P = 0.066
P = 0.907
P = 0.574
Months
Months
Months
Figure S3

## Slide 4
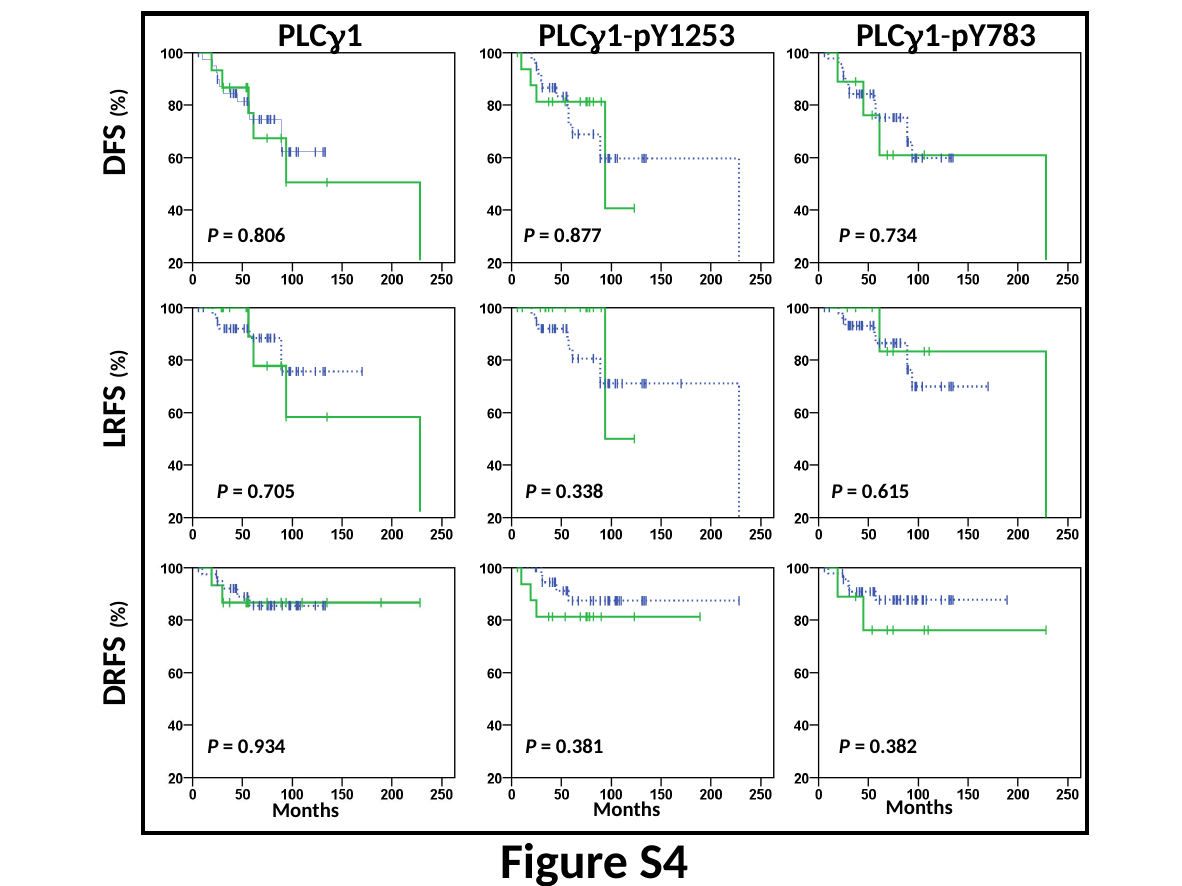

PLCγ1
PLCγ1-pY1253
PLCγ1-pY783
DFS (%)
P = 0.806
P = 0.877
P = 0.734
LRFS (%)
P = 0.705
P = 0.338
P = 0.615
DRFS (%)
P = 0.934
P = 0.381
P = 0.382
Months
Months
Months
Figure S4

## Slide 5
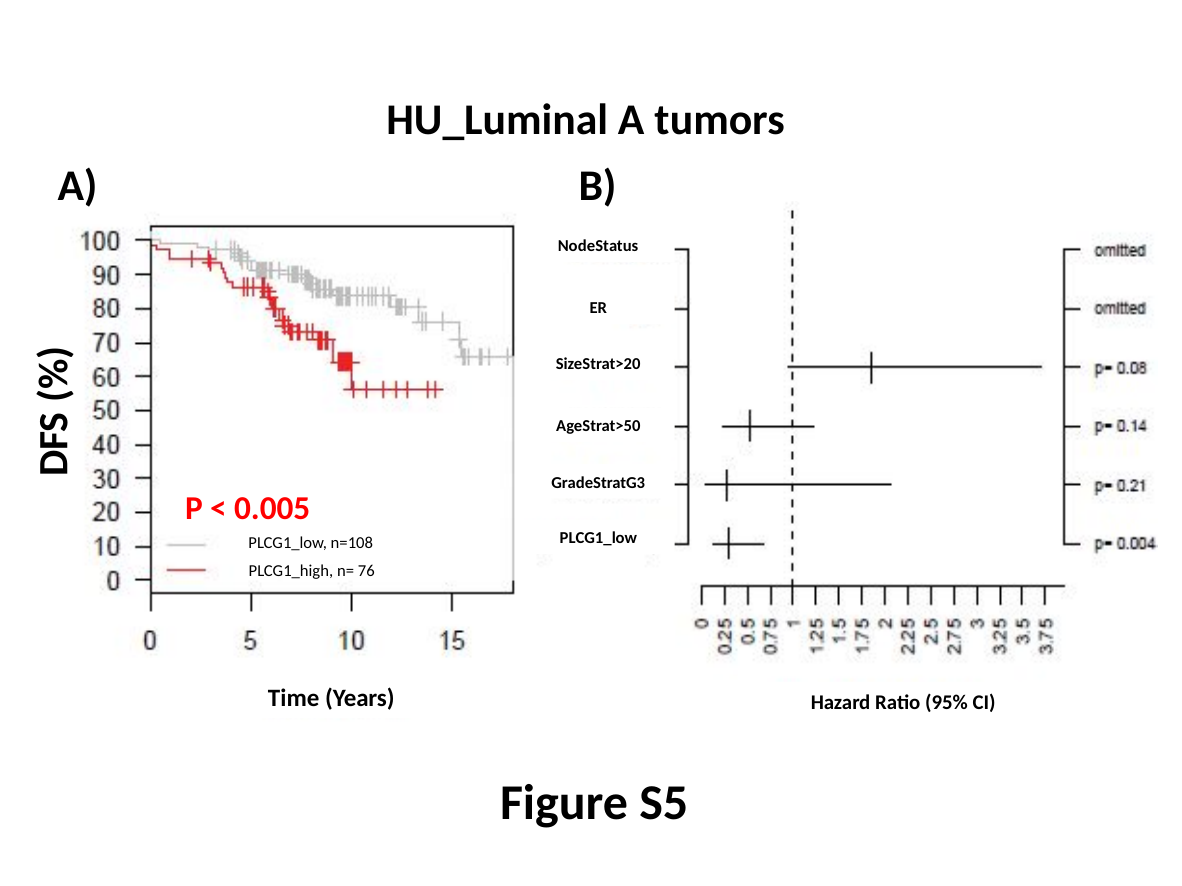

HU_Luminal A tumors
A)
B)
Hazard Ratio (95% CI)
NodeStatus
ER
SizeStrat>20
AgeStrat>50
GradeStratG3
PLCG1_low
P < 0.005
PLCG1_low, n=108
PLCG1_high, n= 76
DFS (%)
Time (Years)
Figure S5

## Slide 6
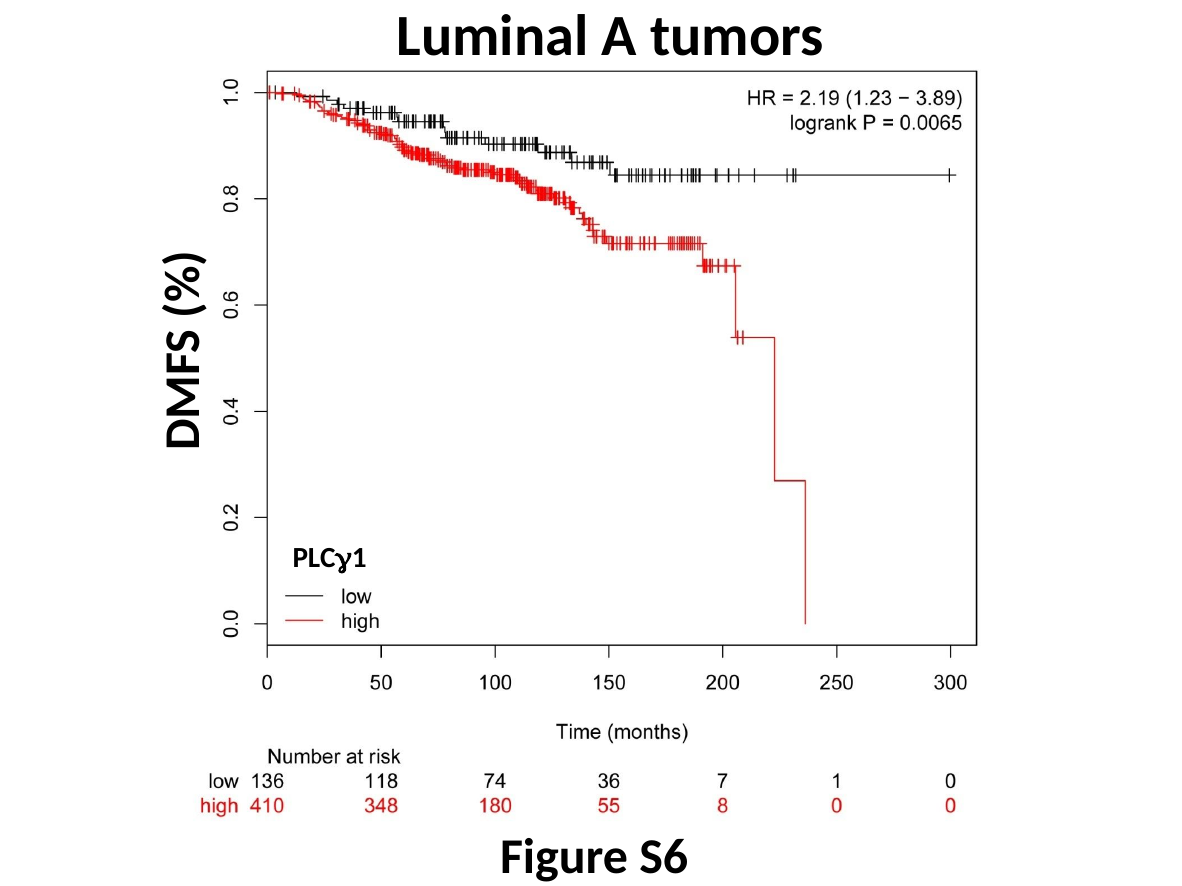

PLCg1
DMFS (%)
Luminal A tumors
Figure S6
